# A review on angio-fibrotic pathways driving cancer potential in oral submucous fibrosis

**DOI:** 10.6026/973206300220610

**Published:** 2026-02-28

**Authors:** Sneha Masne Deshpande, Sonali Khatri, Pawan Rebello, Prachi Gholap, Anupa Roshan Shetty, Sheetal Choudhari, Pankaj Shrikant Deshpande

**Affiliations:** 1 Department of Oral Pathology and Microbiology, Bharati Vidyapeeth (Deemed to be University) Dental College and Hospital, Belapur, Navi Mumbai, Maharashtra, India; 2 Department of Oral Pathology and Microbiology, Genesis Institute of Dental Sciences and Research, Firozpur, Punjab, India; 3 Department of Oral & Maxillofacial Pathology & Oral Microbiology, Sardar Patel Post Graduate Institute of Dental and Medical Sciences, Lucknow, Uttar Pradesh, India; 4 Department of Prosthodontics & Crown and Bridge, Bharati Vidyapeeth (Deemed to be University) Dental College and Hospital, Belapur, Navi Mumbai, Maharashtra, India; 5 Department of Periodontology, Bharati Vidyapeeth (Deemed to be University) Dental College and Hospital Navi Mumbai, Navi Mumbai, Maharashtra, India; 6 Department of Oral Pathology and Microbiology, YMT Dental College and Hospital, Kharghar, Navi Mumbai 400089, India; 7 Department of Civil Engineering, Sidus Buildcon India Private Limited, Navi Mumbai, Maharashtra, India

**Keywords:** Oral submucous fibrosis, areca nut, angiogenesis, fibrosis, VEGF, HIF-1α, oral squamous cell carcinoma (OSCC)

## Abstract

Oral Submucous Fibrosis (OSF) is a chronic, progressive, potentially malignant disorder associated with areca-nut exposure, characterized by persistent inflammation, extracellular matrix accumulation, and fibrotic restriction of oral function. This review highlights how fibrosis-driven vascular compromise and sustained hypoxia generate a tumor-promoting microenvironment that underlies malignant transformation. Dysregulated fibroblast activation, abnormal matrix remodeling, impaired angiogenesis, and chronic inflammatory signaling collectively promote stromal stiffness, oxidative stress, epithelial instability, and EMT-like changes. These interconnected angio-fibrotic and hypoxic mechanisms facilitate progression from fibrosis to epithelial dysplasia and ultimately oral squamous cell carcinoma. Understanding this pathogenic network provides a framework for early risk stratification and supports development of targeted anti-fibrotic and anti-angiogenic therapeutic strategies in OSF.

## Background:

**Figure 1:**
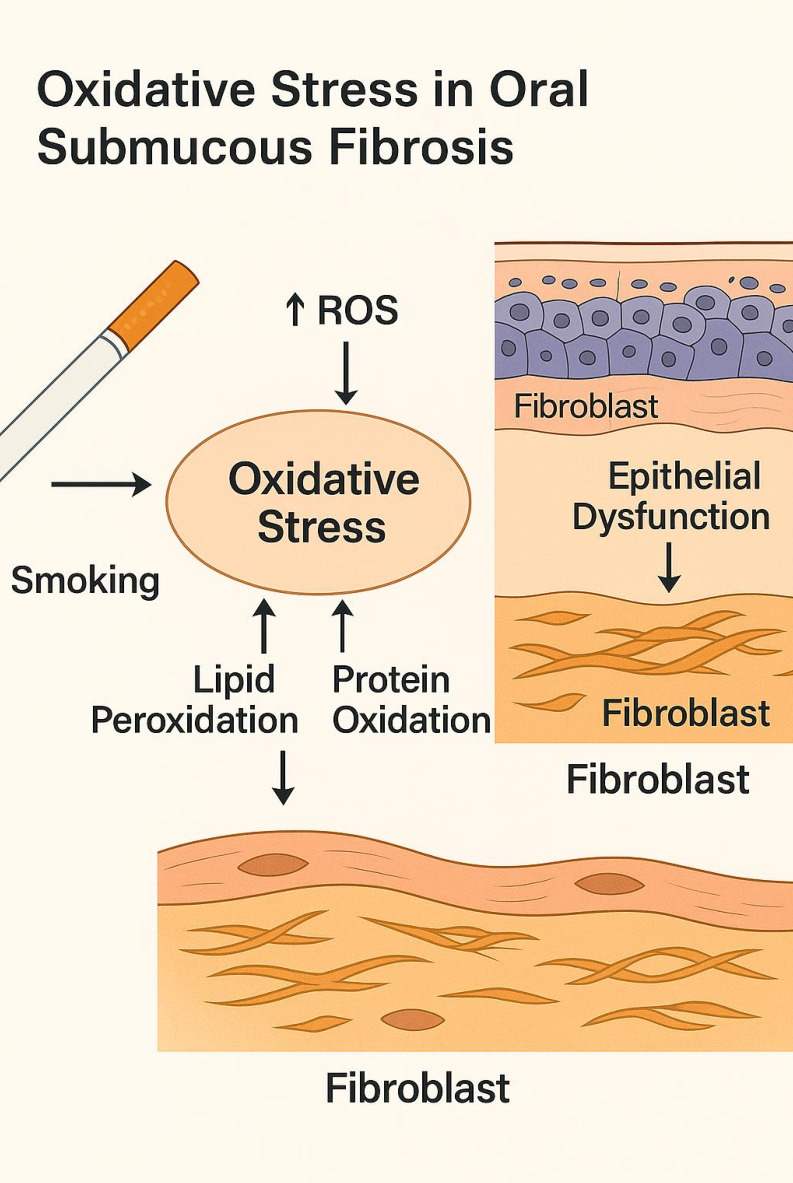
Oxidative stress in oral submucous fibrosis - Reactive oxygen species (ROS) generated by areca-nut metabolites and smoking contribute to lipid peroxidation, protein damage, and fibroblast-mediated fibrogenesis.

Oral Submucous Fibrosis (OSF) is a chronic, progressive, potentially malignant disorder strongly associated with habitual areca-nut chewing, particularly in South and Southeast Asia [1]. Clinically, OSF presents with mucosal blanching, stiffness, burning sensation and progressive reduction in mouth opening due to excessive collagen deposition and reduced tissue elasticity [2]. Histopathologically, OSF is characterized by epithelial atrophy, juxta- epithelial inflammation, hyalinized collagen bundles, and compromised vascularity, reflecting advanced fibrosis and chronic tissue hypoxia [3]. The malignant transformation rate of OSF ranges from 7% to 13%, highlighting its importance as a high-risk oral potentially malignant disorder [4]. Understanding the interactions between fibrogenesis, hypoxia, angiogenesis, oxidative stress, and immune dysregulation is essential to elucidate the mechanisms driving OSF progression toward oral squamous cell carcinoma (OSCC) [5]. Figure 1 illustrate the schematic diagram represents Reactive oxygen species (ROS) generated by areca-nut metabolites and smoking contribute to lipid peroxidation, protein damage, and fibroblast-mediated fibrogenesis. Therefore, it is of interest to report Angio-Fibrotic Pathways Driving Cancer Potential in Oral Submucous Fibrosis.

## Pathogenesis:

## Areca nut, fibrosis and molecular drivers:

**Figure 2:**
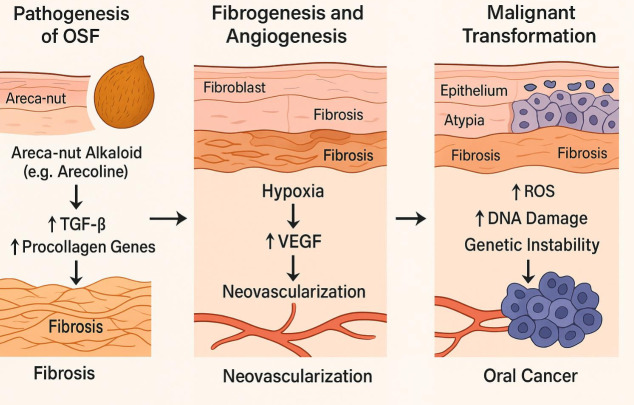
Molecular pathway from OSF to oral cancer - Areca-nut-induced fibrosis triggers chronic hypoxia, HIF-1α activation, VEGF-mediated angiogenesis, and genetic instability that collectively promote malignant transformation.

Areca nut constituents such as arecoline, arecaidine and polyphenols stimulate fibroblast proliferation, upregulate collagen synthesis, and inhibit collagenase activity, resulting in excessive extracellular matrix accumulation in OSF [1]. Arecoline induces TGF-β overexpression, which drives myofibroblast differentiation, enhances ECM cross-linking, and promotes persistent fibrogenesis [2]. Copper presents in areca nut upregulates lysyl oxidase (LOX), increasing collagen cross-linking and tissue stiffness and thereby accelerating fibrosis progression [3]. Areca nut metabolites generate reactive oxygen species (ROS), contributing to lipid peroxidation, DNA strand breaks, and oxidative stress- mediated mutagenesis [4]. Nitrosation of areca alkaloids produces carcinogenic N-nitrosamines that further enhance genomic instability and epithelial vulnerability [1, 4]. Figure 2 illustrate the schematic diagram represents Areca-nut-induced fibrosis triggers chronic hypoxia, HIF-1α activation, VEGF-mediated angiogenesis, and genetic instability that collectively promote malignant transformation. Progressive fibrosis compromises microvascular perfusion, resulting in chronic tissue hypoxia that stabilizes hypoxia-inducible factor-1α (HIF-1α), a central regulator of angiogenesis, glycolytic shift, and pro-survival pathways [5]. The interplay of TGF-β signaling, LOX activation, oxidative stress, and hypoxia forms a self-reinforcing loop that perpetuates collagen deposition and primes the OSF microenvironment for malignant transformation [2, 5].

## Fibrogenesis and hypoxia:

**Figure 3:**
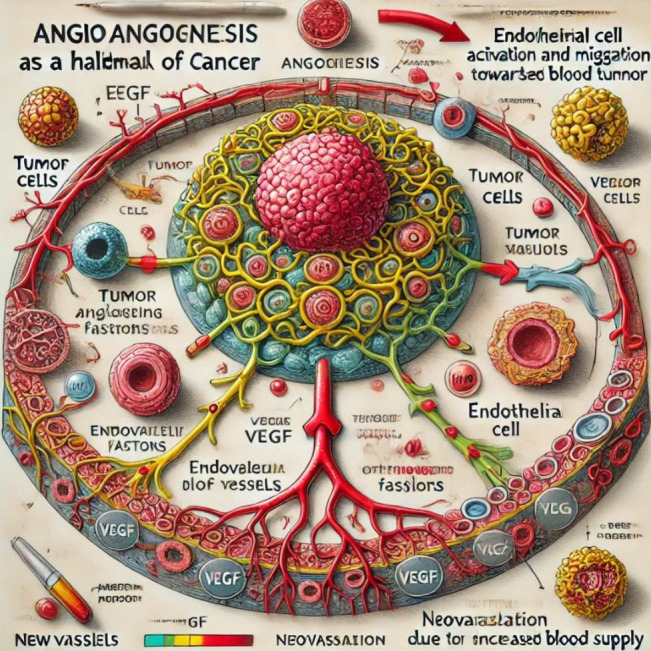
Tumor-induced angiogenesis - Tumor cells secrete VEGF and pro-angiogenic mediators that stimulate endothelial activation, migration, and neovascularization to support tumor progression.

Progressive extracellular matrix (ECM) deposition disrupts the microvascular architecture, resulting in impaired tissue perfusion and the development of chronic hypoxia [6, 8]. Hypoxic stress promotes stabilization of hypoxia-inducible factor-1 alpha (HIF-1α), a key transcriptional regulator that enhances the expression of angiogenic mediators, including vascular endothelial growth factor (VEGF), and contributes to metabolic reprogramming within the diseased mucosa [7, 8]. These hypoxia-driven molecular adaptations support epithelial-mesenchymal transition (EMT), cellular survival under low oxygen tension, and dysplastic progression [2, 6]. Figure 3 illustrates the schematic diagram represents Tumor-induced angiogenesis. Tumor cells secrete VEGF and related pro-angiogenic mediators that activate endothelial cells, promote their migration and sprouting, and drive neovascularization, thereby sustaining tumor growth and facilitating progression (Figure 3). Tumor cells secrete VEGF and pro-angiogenic mediators that stimulate endothelial activation, migration, and neovascularization to support tumor progression.

## Angiogenesis in OSF:

Friend or Foe?

**Figure 4:**
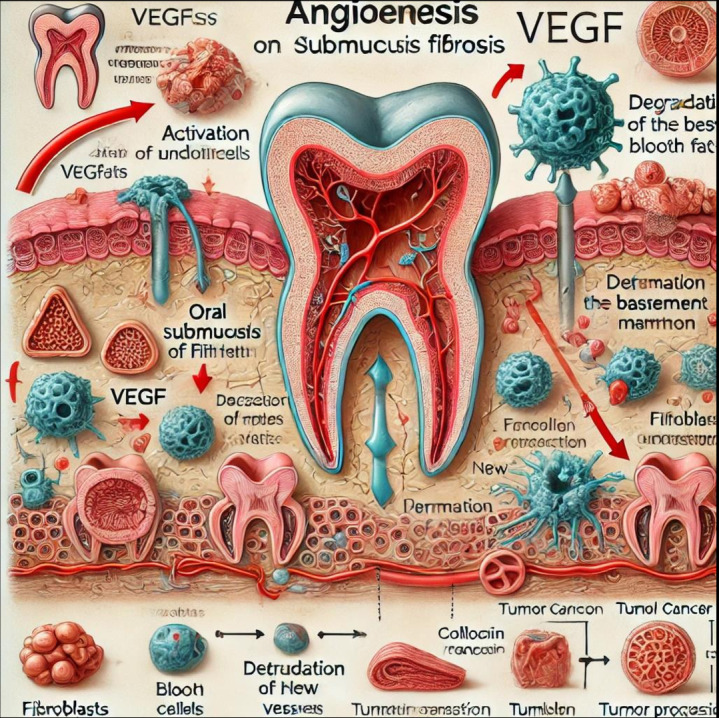
VEGF-mediated angiogenesis in OSF - Hypoxia-driven VEGF signaling promotes formation of new but structurally abnormal blood vessels within fibrotic oral mucosa.

Chronic fibrosis in OSF progressively reduces microvascular density, leading to sustained tissue hypoxia that activates HIF-1α and upregulates VEGF as a compensatory angiogenic response [6]. In early OSF, VEGF-mediated neovascularization may transiently improve oxygen delivery; however, persistent fibrosis interferes with vascular maturation, producing structurally abnormal, leaky and poorly functional blood vessels [5]. Dysregulated angiogenesis increases vascular permeability, facilitates inflammatory cell infiltration, and supports epithelial survival under hypoxic stress [7]. These abnormal neovessels provide nutrients to dysplastic epithelial clusters and enable early invasion, accelerating transition toward oral squamous cell carcinoma [8]. Thus, angiogenesis in OSF begins as an adaptive response but becomes maladaptive, promoting malignant transformation through defective vascular remodeling and hypoxia-driven signaling [6]. Figure 4 illustrate the schematic diagram represents Hypoxia-driven VEGF signaling promotes formation of new but structurally abnormal blood vessels within fibrotic oral mucosa.

## Molecular interplay promoting malignant transformation:

Persistent TGF-β overexpression in OSF drives myofibroblast activation, ECM accumulation, and immune suppression, creating a permissive environment for dysplastic epithelial survival [2]. Fibrosis-associated hypoxia stabilizes HIF-1α, activating transcription of VEGF, GLUT-1, and other hypoxia-responsive genes that enhance angiogenesis, metabolic adaptation, and apoptosis resistance [6]. Hypoxia-driven VEGF signaling generates immature neovessels that increase nutrient supply to dysplastic regions and facilitate early invasion [7]. Oxidative stress and ROS-induced DNA damage accumulate in the epithelium, promoting genomic instability [4]. Senescent fibroblasts secrete SASP mediators such as IL-6, IL-8, and MMPs, which stimulate epithelial proliferation, ECM degradation, and tumor-promoting inflammation [8]. Increased MMP-2 and MMP-9 expression disrupts basement membrane integrity and supports epithelial migration [7]. Together, fibrosis, hypoxia, angiogenic dysregulation, oxidative stress, and fibroblast senescence create a synergistic microenvironment that drives malignant transformation in OSF [5].

## Immune dysregulation and fibroblast senescence:

Chronic inflammation in OSF alters local immune responses by promoting a shift toward immunosuppressive cytokine signaling, largely driven by persistent TGF-β activation [2]. Prolonged exposure of fibroblasts to TGF-β and oxidative stress induces fibroblast senescence, resulting in the accumulation of senescent-associated secretory phenotype (SASP) factors [8]. SASP mediators-including IL-6, IL-8, CXCL12, and MMPs-enhance epithelial proliferation, disrupt normal matrix architecture, and promote a tumor-supportive microenvironment [8]. Senescent fibroblasts also impair antigen presentation and reduce immune surveillance, enabling dysplastic epithelial cells to evade immune-mediated clearance [9]. These combined effects of immune suppression, chronic SASP-mediated inflammation, and matrix degradation facilitate epithelial instability, enhancing the likelihood of malignant transformation in OSF [8, 9].

## Biomarkers and diagnostic tools:

Several molecular biomarkers are under investigation to identify OSF patients at high risk of malignant transformation, including VEGF, HIF-1α, LOX, MMP-2, MMP-9, and inflammatory cytokines reflecting hypoxia-driven angiogenesis and ECM remodeling [6]. Elevated VEGF and HIF-1α correlate with disease severity and hypoxic adaptation [7]. LOX overexpression indicates collagen cross-linking and increased tissue stiffness associated with advanced fibrosis [3]. MMP-2 and MMP-9 reflect active ECM degradation and invasion-associated remodeling [8]. Emerging diagnostic technologies include salivary biomarker panels, quantitative immunohistochemistry, digital pathology, and AI-assisted image analysis for objective dysplasia assessment [9]. These tools may support early detection and risk prediction in OSF [9].

## Therapeutic approaches and future directions:

Conventional OSF management includes habit cessation, nutritional support, physiotherapy, and intralesional corticosteroids or hyaluronidase [2]. Anti-fibrotic agents such as pirfenidone and TGF-β pathway inhibitors show promise in reducing ECM deposition [6]. VEGF-targeted and anti-angiogenic therapies may counteract hypoxia-driven vascular abnormalities [7]. Antioxidants and chemopreventive agents such as curcumin and lycopene may reduce oxidative DNA damage [4]. Senolytic therapies targeting senescent fibroblasts are emerging strategies to suppress SASP-mediated inflammation [8]. Advances in AI-based histopathology and biomarker profiling may enable personalized risk stratification [9]. Future research should emphasize longitudinal and multicentric cohort studies to validate biomarkers and therapeutic targets [5].

## Limitations and research gaps:

OSF literature shows methodological variability, including heterogeneous diagnostic criteria and staging systems, limiting comparability across studies [7]. Many studies use cross-sectional or small sample designs, restricting assessment of disease progression [6]. Longitudinal multicentric data linking molecular alterations from early OSF to OSCC remain scarce [6]. Biomarker validation in large populations is limited [8]. Advanced tools such as digital pathology and AI-based dysplasia grading remain underutilized [9]. Well-designed prospective studies integrating molecular and clinical outcomes are required to improve early cancer detection in OSF [5].

## Conclusion:

OSF creates a pro-carcinogenic microenvironment through the combined effects of fibrosis, hypoxia, angiogenic imbalance, oxidative stress, and immune dysregulation, largely driven by areca-nut exposure. Integrating molecular biomarkers with digital pathology and AI-based diagnostics can enhance early risk prediction and prevention of OSF-associated oral cancer, warranting multicentric longitudinal validation studies.

## Advancement to knowledge:

This review presents an integrated angio-fibrotic-hypoxic model explaining malignant transformation in Oral Submucous Fibrosis, emphasizing vascular dysfunction and hypoxia as central drivers rather than secondary effects. It links fibrosis, angiogenesis, immune dysregulation, and fibroblast senescence into a single mechanistic framework and proposes biologically aligned biomarkers for early risk prediction and targeted intervention.

## Funding:

No external funding was received for this review.
